# Assessing the Effects of Bakhour on Pulmonary Functions in Young Adults in Saudi Arabia

**DOI:** 10.7759/cureus.89367

**Published:** 2025-08-04

**Authors:** Lara M Aljohani, Shahabuddin T Shaikh, Amen A Bawazir, Laraib M Chishtee, Maria A Alrafi, Bashayer K Alskait, Kenaz A Alzughibi, Tharaa H Alkhudairi, Maheen S Shaikh

**Affiliations:** 1 Physiology Department, AlMaarefa University, Riyadh, SAU; 2 Physiology Department, College of Medicine, AlMaarefa University, Riyadh, SAU; 3 Community Health Department, College of Medicine, AlMaarefa University, Riyadh, SAU; 4 Physiology Department, Al Faisal University, Riyadh, SAU

**Keywords:** bakhour, incense, lung function, pulmonary function tests, respiratory health

## Abstract

Background

Bakhour, a traditional incense commonly used in Saudi Arabia, is widely burned indoors, raising concerns about its impact on pulmonary health. Studies on the respiratory effects of Bakhour are limited.

Objective

This study aims to assess the effects of Bakhour exposure on pulmonary function in young adults in Saudi Arabia, evaluating prevalence, pulmonary function test (PFT) comparisons, and correlations with demographic factors.

Methods

A descriptive cross-sectional study was conducted at Al-Maarefa University, Riyadh, from November 2022 to August 2023. A total of 130 voluntary participants aged 18 and above were recruited. Standard spirometry tests were performed, and data were analyzed using SPSS v28 (IBM Corp., Armonk, NY, US).

Results

Bakhour use was more common among men (86.2%) as compared to women (69.2%) and smokers (94.1%) as compared to non-smokers (71.9%). No significant relationship was found between Bakhour use and pulmonary function; however, age, gender, and BMI significantly influenced lung function.

Conclusion

While Bakhour use was prevalent, it was not significantly associated with poor lung function. Future research should explore larger sample sizes and better exposure assessments.

## Introduction

Indoor incense burning has been a cultural practice for centuries, and it is often used for religious, spiritual, or aesthetic purposes. Bakhour is a type of traditional indoor incense that is commonly used in the Middle East and North Africa for various purposes, including regular use, special occasions, social gatherings, and in places of worship [1]. However, its potential impact on human health, specifically on pulmonary function, remains an area of concern. It is one of the common sources of indoor air pollution to which individuals are exposed on a regular basis in most homes in Saudi Arabia [2].

Bakhour can vary in composition; it usually contains a variety of natural ingredients, including agarwood (oud), woodchips, musk, and sandalwood soaked in scented oils [3]. Due to its slow and incomplete combustion. Bakhour burning produces continuous smoke, which generates multiple air pollutants, such as toxic gases and chemical particles, including polycyclic aromatic hydrocarbons, carbon monoxide, isoprene, and benzene, all of which facilitate indoor accumulation, especially under inadequate ventilation [4].

Bakhour is commonly used in many cultures and for daily use in Saudi Arabia. Earlier studies have proven the association of indoor incense with various health burdens; however, a lack of research on Bakhour use in the Saudi Arabian population has been demonstrated [5]. This study aims to explore the association between Bakhour exposure and pulmonary function test (PFT) results in young healthy individuals in Riyadh, Saudi Arabia. The findings of this study will significantly contribute to the awareness of health risks related to indoor incense exposure, in addition to informing public health policies and guidelines. This study aimed to assess the effects of Bakhour on pulmonary function in young adults in Riyadh, Saudi Arabia, focusing on prevalence, PFT comparisons, and correlations with demographic factors.

## Materials and methods

Study design and settings

This study was designed as a descriptive cross-sectional investigation to examine the relationship between Bakhour usage and lung function among students at the College of Medicine, AlMaarefa University, Riyadh, Saudi Arabia. Data collection occurred between November 2022 and August 2023. The study was approved by the Institutional Review Board (IRB No. IRB23-060) at AlMaarefa University.

Sample size

A combination of convenience sampling and sample size estimation using the Raosoft calculator was employed. Convenience sampling was chosen due to the accessibility and availability of students within the university setting. To enhance validity, the Raosoft sample size calculator (http://www.raosoft.com/samplesize.html) was used with a 95% confidence level and 5% margin of error, resulting in a required sample of 130 students. An initial pilot study involving 15 students was conducted to refine the data collection process. The final sample included both male and female students. Participation was voluntary, and informed consent was obtained from all participants prior to data collection.

Subjects

Participants were students from AlMaarefa University, aged 18 years or older, and enrolled in various medical specialties. Medical conditions were based on participants’ self-reports; individuals with cardiovascular diseases, respiratory diseases, thoracic deformities, and those experiencing acute symptoms at the time of data collection were excluded, as such conditions could temporarily alter PFT results and affect data validity.

Data collection

All data were collected through structured face-to-face interviews conducted before the PFT. Information collected included the frequency of Bakhour exposure, categorized as: Minimum (at most once monthly/occasional use); Average (once to four times weekly); Extensive (once or more daily).

Participants were also asked about duration and quantity of exposure, number of years exposed, smoking habits, and any pre-existing or current respiratory symptoms such as cough or nasal congestion.

Spirometry lung function test

The spirometry lung function test was conducted by trained personnel using a new Spirolab® spirometer (Medical International Research (MIR), Rome, Italy; portable desktop model with oximetry option and built-in thermal printer). Tests followed standard protocols. Participants were advised to avoid caffeine and strenuous activity for at least 30 minutes before testing. Each participant performed up to three attempts to ensure measurement reliability.

Data analysis

Data were entered in Microsoft Excel 2023 (Microsoft Corporation, Redmond, WA, US) and analyzed using SPSS Version 28 (IBM Corp., Armonk, NY, US). Frequencies and percentages were used to summarize participant characteristics. The chi-square test was used to assess associations between sociodemographic factors and Bakhour exposure or pulmonary function parameters. Logistic regression was used to evaluate the relationship between PFT results (forced vital capacity (FVC), forced expiratory volume in 1 second (FEV1)/FVC ratio, PEF) and categorical exposure levels, with p < 0.05 considered statistically significant.

## Results

Table 1 shows the sociodemographic characteristics of the participants. The data presents information on various variables among the participants, including age, gender, BMI (body mass index), smoking status, Bakhour use, and intensity of use. The majority of the participants were in the age group 20-22 years (46.2%), with normal BMI (51.1%), non-smokers (73.8%), Bakhour users (77.7%), with minimum use (38.5%). However, gender was equal (50% for each male and female).

**Table 1:** Sociodemographic characteristics of the participants Includes age, gender, BMI (body mass index), smoking status, Bakhour (BR) use, and intensity of BR exposure

Variables	Categories	No.	%
Age	≤ 19 years	39	30.0
	20-22 years	60	46.2
	≥23 years	31	23.8
Gender	male	65	50.0
	female	65	50.0
BMI	normal	67	51.5
	underweight	13	10.0
	overweight	33	25.4
	obese	17	13.1
Smoking status	non-smoker	96	73.8
	smoker	34	26.2
Bakhoor use	non-user	29	22.3
	BR user	101	77.7
Intensity of use	non-user	29	22.3
	minimum use	50	38.5
	average use	31	23.8
	extensive use	20	15.4

Table 2 summarizes the relationship between Bakhour exposure and sociodemographic characteristics. The results indicate that Bakhour exposure is significantly associated with gender and smoking status. Specifically, men appear more likely to be exposed to Bakhour (86.2%) as compared to women (69.2%) (p = 0.020). Smokers are more likely to be exposed (94.1%) compared to non-smokers (71.9%) with a significant p-value (p = 0.007). However, age and BMI categories do not appear to be significantly associated with Bakhour exposure.

**Table 2:** Bakhour exposure in relation to sociodemographic characteristics Shows the association between Bakhour (BR) use and age, gender, BMI, smoking, and p-values for statistical significance

Variables	Categories	Non-users		Users		
		No.	%	No.	%	P-value
Age	≤19 years	10	25.6	29	74.4	0.602
	20-22 years	11	18.3	49	81.7	
	≥23 years	8	25.8	23	74.2	
Gender	male	9	13.8	56	86.2	0.020
	female	20	30.8	45	69.2	
BMI	normal	9	13.8	56	86.2	.858
	underweight	20	30.8	45	69.2	
	overweight	9	13.8	56	86.2	
	obese	20	30.8	45	69.2	
Smoking status	non-smoker	27	28.1	69	71.9	0.007
	smoker	2	5.9	32	94.1	

Table 3 presents the association between poor forced vital capacity (FVC), forced expiratory volume in one second (FEV1), FEV1/FVC ratio, and peak expiratory flow (PEF) with various sociodemographic factors. The table categorizes participants by age, gender, body mass index (BMI), smoking status, Bakhour (BR) use, and intensity of smoking. Regarding age, individuals aged 20-22 show the highest percentages of poor lung function across all parameters, while those aged <19 or ≥23 demonstrate slightly lower rates. Gender-wise, females generally exhibit higher percentages of poor lung function compared to males. BMI classification reveals that individuals classified as underweight or obese tend to have higher percentages of poor lung function, especially in terms of the FEV1/FVC ratio. Smoking status does not show a clear pattern, with both smokers and non-smokers displaying varying percentages of poor lung function across the parameters. Significant p-values indicate associations between certain sociodemographic factors and poor lung function. Notably, age groups, gender, and BMI categories show significant associations with poor FVC (p-value =0.012, 0.003, respectively) and FEV1 (p-value =0.008), while the FEV1/FVC ratio is significantly associated with age and smoking status. PEF is significantly associated with age (p-value =0.028) and BMI categories. These findings underscore the importance of considering sociodemographic factors when assessing lung function, with age, gender, BMI, and smoking status being particularly influential. Overall, the table suggests that age, smoking, and potentially BMI play a role in lung function.

**Table 3:** The association between poor FVC, FEV1, FEV1/FVC ratio, and PEF with sociodemographic factors Presents FVC (forced vital capacity), FEV1 (forced expiratory volume in 1 second), FEV1/FVC ratio, and PEF (peak expiratory flow) across demographic and exposure variables

Variable	Category	FVC		FEV1		FEV1/FVC ratio		PEF	
		No	%	No	%	No	%	No	%
Age	<19	27	69.2	31	79.5	24	61.5	36	92.3
	20-22	48	80.0	54	90.0	48	80.0	60	100.0
	≥23	25	80.6	24	77.4	21	67.7	31	100.0
Gender	Male	44	67.7	51	78.5	46	70.8	62	95.4
	Female	56	86.2	58	89.2	47	72.3	65	100.0
BMI	Normal	57	85.1	61	91.0	48	71.6	67	100.0
	Underweight	12	92.3	12	92.3	8	61.5	13	100.0
	Overweight	23	69.7	26	78.8	23	69.7	31	93.9
	Obese	8	47.1	10	58.8	14	82.4	16	94.1
Smoking	No	71	74.0	78	81.2	68	70.8	93	96.9
	Yes	29	85.3	31	91.2	25	73.5	34	100.0
BR Use	No	19	65.5	22	75.9	21	72.4	28	96.6
	Yes	81	80.2	87	86.1	72	71.3	99	98.0
Intensity	No	19	65.5	22	75.9	21	72.4	28	96.6
	Minimum	37	74.0	40	80.0	37	74.0	48	96.0
	Average	26	83.9	29	93.5	24	77.4	31	100.0
	Extensive	18	90.0	18	90.0	11	55.0	20	100.0

Table 4 presents the association between Bakhour users and PFT parameters, specifically FVC, FEV1, FEV1/FVC ratio (RATIO), and peak expiratory flow (PEF). Overall, while there seem to be slightly higher percentages of non-users with decreased FVC and FEV1 compared to users, these differences are not statistically significant. Similarly, there is no significant difference in the distribution of the FEV1/FVC ratio and PEF between non-users and users of Bakhour.

**Table 4:** Association between Bakhour users and pulmonary function test parameters Compares FVC (forced vital capacity), FEV1 (forced expiratory volume in 1 second), FEV1/FVC ratio, PEF (peak expiratory flow) between BR (Bakhour) users and non-users; includes p-values

Variable	Category	All		Non-users		Users		
		No.	%	No.	%	No.	%	P-value
FVC	Decreased	100	76.9	19	65.5	81	80.2	.098
	Normal	30	23.1	10	34.5	20	19.8	
FEV1	Decreased	109	83.8	22	75.9	87	86.1	.185
	Normal	21	16.2	7	24.1	14	13.9	
FEV1/FVC ratio	Decreased	93	71.5	21	72.4	72	71.3	.906
	Normal	37	28.5	8	27.6	29	28.7	
PEF	Decreased	127	97.7	28	96.6	99	98.0	.643
	Normal	3	2.3	1	3.4	2	2.0	

Table 5 presents the results of a logistic regression model analyzing the association between PFT parameters (FVC, FEV1/FVC ratio, and PEF) and their categories. The findings suggest there's no significant association (p-value in all is >0.05) between decreased FVC (OR=1.845, 95%CI=.526-6.469), FEV (OR=11.249, 95%CI=.293-5.329), FEV1/FVC ratio category (OR=0.936, 95% CI=.347-2.520), and the PEF category (OR=1.226, 95% CI=.095-15.810) with the outcome being studied, which is Bakhour use. Overall, none of the PFT parameters show statistically significant associations with the outcome being studied. However, caution should be exercised, especially considering the wide confidence intervals, which indicate uncertainty in the estimates. Additional analysis or larger sample sizes might be needed to draw more conclusive findings.

**Table 5:** Logistic regression model with the pulmonary function test parameters Shows OR (odds ratio), CI (confidence interval), and p-values for the association between BR (Bakhour) use and decreased lung function parameters

Variable	Category	OR	95%CI	P-value
FVC	Normal	Reference	-	-
	Decreased	1.845	.526-6.469	.339
FEV1	Normal	Reference	-	-
	Decreased	1.249	.293-5.329	.764
FEV1/FVC ratio	Normal	Reference	-	-
	Decreased	.936	.347-2.520	.895
PEF	Normal	Reference	-	-
	Decreased	1.226	.095-15.810	.876

The linear regression model analyzes the relationship between lung function (FEV1_2) and various factors like age, gender, BMI, smoking, and Bakhour use and its intensity. Results show that only the BMI category significantly predicts lung function, with higher BMI associated with decreased lung function. Other variables, such as age, gender, smoking, and Bakhour use and its intensity, do not significantly influence lung function in this model. The standardized coefficients (Beta) indicate the relative strength of each variable's effect on lung function, with the BMI category having a relatively strong inverse effect, as seen in Table 6.

**Table 6:** Linear regression model analyses the relationship between lung function (FEV1_2) and sociodemographic factors Analyzes effects of age, gender, BMI, smoking, BR (Bakhour) use, and intensity on FEV1_2; includes beta coefficients and P-values

Variable	Unstandardized Coefficients		Standardized Coefficients	t	P-value
	B	Std. Error	Beta		
Age	.001	.044	.003	.030	.976
Gender	.078	.069	.106	1.143	.255
BMI	-.077	.029	-.239	-2.701	.008
Smoking	.086	.074	.103	1.173	.243
BR Use	.031	.115	.035	.273	.785
Intensity	.039	.047	.103	.817	.416

Table 7 shows a regression model predicting the FVC, FEV1, and FEV1/FVC ratio in correlation with various demographic characteristics. Significant associations are observed between FEV and gender (marginally significant with OR of 0.333 (95% CI: 0.110-1.010). BMI, specifically obesity, has a significantly higher FEV (5.7 times, 95% CI: 1.589-20.448 and p-value = 0.008), as well as with FEV1, with OR five times higher when compared with normal BMI (5.711, 95% CI: 1.424-22.909, and p-value 0.014). On the other hand, significant associations were observed between FVC/FEV1 and age, specifically the 20-22 age group (OR= 0.377 (95% CI: 0.147-0.970, a p-value of 0.043) compared to those aged ≤19 years, suggesting that individuals in this age group are less likely to have lower FEV compared to those aged ≤19. No significant associations were found for smoking status, Bakhour use, and intensity of use.

**Table 7:** Regression model predicting the correlation between FEV1/FVC and demographic characteristics Presents ORs (odds ratios), CIs (confidence intervals), and p-values for factors predicting a decreased forced expiratory volume in 1 second (FEV1)/forced vital capacity (FVC) ratio

Variable	Category	FVC		FEV1		FEV1/FVC Ratio	
		OR (95%CI)	P-value	OR (95%CI)	P-value	OR (95%CI)	P-value
Age	<19	R	-	R	-	R	-
	20-22	.519(.180-1.493)	.224	.422(.119-1.498)	.182	.377(.147-.970)	.043
	≥23	.558(.152-2.040)	.378	1.343(.102-1.278)	.665	.841(.295-2.400)	.746
Gender	Male	R	-	R	-	R	-
	Female	.333(.110-1.010)	.052	.360(.102-1.278)	.114	.633(.257-1.560)	.321
BMI	Normal	R	-	R	-	R	-
	Underweight	.598(.064-5.551)	.651	.703(.066-7.464)	.770	1.390(.357-5.423)	.635
	Overweight	1.731(.580-5.161)	.325	1.710(.474-6.164)	.412	.943(.352-2.527)	.906
	Obese	5.700(1.589-20.448)	.008	5.711(1.424-22.909)	.014	.444(.107-1.846)	.264
Smoking	No	R	-	R	-	R	-
	Yes	.479(.144-1.588)	.229	.357(.082-1.551)	.169	.663(.249-1.768)	.412
BR Use	No	R	-	R	-	R	-
	Yes	.255(.043-1.514)	.133	.514(.078-3.382)	.488	2.476(.674-9.103)	.172
Intensity	No	R	-	R	-	R	-
	Minimum	1.826 (.324-10.307)	.495	1.144(.181-7.224)	.886	.428(.131-1.399)	.160
	Average	1.126(.175-7.247)	.901	.423(.047-3.813)	.443	.346(.094-1.278)	.111

Summary of results

The study included an equal distribution of male and female participants (50% each), predominantly aged 20-22 years (46.2%). Most participants had a normal BMI (51.1%) and were non-smokers (73.8%). Bakhour use was reported by 77.7% of the participants, with minimal intensity being the most common pattern of use (38.5%). Bakhour exposure showed significant associations with gender and smoking status, with males (86.2%) and smokers (94.1%) more likely to report exposure compared to females (69.2%) and non-smokers (71.9%), respectively (p = 0.020 and p = 0.007). However, no significant relationships were found between Bakhour exposure and age or BMI categories. Regarding lung function parameters, age, gender, and BMI were significantly associated with poorer FVC and FEV1. Specifically, participants aged 20-22 years, females, and those classified as underweight or obese exhibited higher percentages of impaired lung function (p-values ranging from 0.003 to 0.012). The FEV1/FVC ratio was significantly linked to age and smoking status, while PEF showed significant associations with age and BMI (p < 0.05). When comparing Bakhour users and non-users, no statistically significant differences were observed across lung function parameters, including FVC, FEV1, FEV1/FVC ratio, and PEF. Although these findings suggest that Bakhour use may not have a measurable impact on these pulmonary measures within this sample, the data contribute valuable information to an area with limited prior research. Logistic regression analysis further supported the lack of significant associations between Bakhour use and decreased lung function parameters (all p-values > 0.05), while also indicating the need for cautious interpretation due to wide confidence intervals. Linear regression identified BMI as the only significant predictor of decreased lung function, with no significant effects attributed to age, gender, smoking, or Bakhour use and intensity. Finally, the regression model examining demographic characteristics revealed significant associations of lung function with gender and BMI. Specifically, females showed a marginally lower FEV (OR=0.333, 95% CI: 0.110-1.010), and obesity was linked to significantly higher odds of altered FEV and FEV1 (OR=5.7 and OR=5.7, respectively; p < 0.015). Additionally, individuals aged 20-22 years were less likely to exhibit lower FEV1/FVC ratios compared to those aged ≤19 years. No significant associations were found with smoking status, Bakhour use, or intensity. Overall, these findings highlight important demographic influences on lung function and suggest that while Bakhour use was not significantly associated with lung function impairment in this cohort, further research with larger samples may help clarify its potential effects.

## Discussion

The study on the impact of Bakhour use among students, predominantly aged 20-22, at AlMaarefa University in Saudi Arabia provides valuable insights into the cultural and health implications of this traditional practice. Bakhour, an aromatic substance widely used in Middle Eastern cultures, has a high prevalence of use among the students who participated in our research, with 77.7% of participants reporting regular exposure. This high rate aligns with the deep-rooted cultural significance of Bakhour in Saudi Arabia, where it is commonly used in various ceremonial and everyday contexts [5].

The widespread use of Bakhour may also be influenced by its aromatic appeal and perceived quality, which is often evaluated using traditional and scientific methods [3]. Additionally, the chemical complexity and potential health risks associated with raw Arabian incense have been highlighted in prior research, which identified a range of hazardous constituents in Bakhour smoke [4].

Despite the widespread use of Bakhour reported in our study, there was no statistically significant association found between Bakhour use and poor lung function indicators such as FVC, FEV1, FEV1/FVC ratio, and PEF. This finding does not align with other research [1]; a possible explanation for this discrepancy could be that PFT results are effort-dependent [6].

Age, gender, and BMI were found to significantly influence respiratory function in the study population. Participants aged 20-22 exhibited poorer lung function metrics compared to younger individuals, a finding consistent with existing literature that correlates age with decreased lung function [6]. Similarly, females demonstrated lower FVC and FEV1 compared to males, aligning with prior research indicating gender differences in lung function [7]. BMI also showed a significant association with FVC and FEV1, with obese individuals exhibiting poorer lung function, consistent with existing studies linking obesity to impaired respiratory function [8]. These sociodemographic factors are crucial when evaluating respiratory health, as they may modulate the effects of environmental exposures like Bakhour.

Notably, the study did not find a significant association between smoking status and the measured respiratory parameters. This could be attributed to the relatively small proportion of smokers in the study population or differences in smoking intensity and duration compared to other studies. Aging, as a factor, is also known to contribute to a gradual decline in lung function due to structural and immunological changes in the lungs [9], which may interact with smoking exposure and further complicate interpretations. Lin et al. (2008) highlighted that incense burning produces significant amounts of particulate matter and gaseous pollutants, which can pose substantial respiratory risks [10]. Beyond environmental exposures, the biological burden of smoking on the lungs is well-documented. Notably, Eckhardt et al. (2022) demonstrated that DNA methylation markers of tobacco smoke exposure can predict both lung function impairment and all-cause mortality [11], reinforcing the long-term impact of smoking on respiratory health.

In addition to combustion-related pollutants, the emissions from incense such as Bakhour have been shown to release a variety of harmful air pollutants under controlled conditions, which include particulate matter and volatile organic compounds [12]. These compounds have been linked to respiratory infections and asthma in other studies. Long-term tobacco use has also been associated with cellular aging, with studies such as Andujar et al. (2018) linking smoking to shortened telomere length and accelerated decline in lung function over time [13]. More recently, Nyilas et al. (2022) used advanced MRI techniques to demonstrate that both vaping and smoking can cause detectable changes in lung perfusion, indicating subclinical damage that might not be captured through basic spirometry alone [14].

Although previous studies have indicated that incense smoke, including Bakhour, might contribute to respiratory issues [15], this study did not find a significant impact on the studied respiratory parameters. The potential reasons for this discrepancy include methodological differences, sample characteristics, and the specific Bakhour formulations used.

The study has several limitations that should be considered when interpreting the findings. The cross-sectional design restricts causal inferences, and the reliance on self-reported Bakhour use may not fully capture actual exposure levels. A key limitation of our study is the small sample size, which may have been insufficient to detect small but clinically significant effects of Bakhour use on respiratory health. Additionally, nose clips were not used during spirometry, which may have affected the accuracy of certain pulmonary function measurements. Future research should consider longitudinal designs and objective measures of Bakhour exposure to better understand its potential health impacts. It is also recommended that future studies encompass a wide range of age groups, along with varied frequencies and intensities of Bakhour use. Furthermore, exploring the chemical composition of different Bakhour formulations and their specific effects on respiratory health could provide deeper insights.

The high prevalence of Bakhour use demonstrated in our study highlights the significance of conducting similar research in the Kingdom of Saudi Arabia and other Arab countries. This is particularly important given the limited number of existing pieces of literature on Bakhour use in these regions. Additionally, to our knowledge, there is a lack of similar research conducted in Riyadh, where our study was carried out.

## Conclusions

While Bakhour is widely used among students in Saudi Arabian universities, the study’s findings do not show a significant association between Bakhour use and adverse respiratory outcomes. This could be attributed to the fact that we have utilized a smaller sample size. However, BMI was an important factor influencing lung function. Future research on this topic is crucial, particularly in Gulf countries. To enhance the value of the research, it is essential to increase the sample size and ensure that participants are fully committed to providing accurate and reliable results.
